# Anthrax Toxins in Context of *Bacillus anthracis* Spores and Spore Germination

**DOI:** 10.3390/toxins7083167

**Published:** 2015-08-17

**Authors:** Christopher K. Cote, Susan L. Welkos

**Affiliations:** United States Army Medical Research Institute of Infectious Diseases (USAMRIID), Bacteriology Division, 1425 Porter Street, Fort Detrick, Frederick, MD 21702-5011, USA; E-Mail: susan.l.welkos.civ@mail.mil

**Keywords:** anthrax, *Bacillus anthracis*, spores, anthrax toxins, PA, germination

## Abstract

The interaction of anthrax toxin or toxin components with *B. anthracis* spores has been demonstrated. Germinating spores can produce significant amounts of toxin components very soon after the initiation of germination. In this review, we will summarize the work performed that has led to our understanding of toxin and spore interactions and discuss the complexities associated with these interactions.

## 1. Toxins Are Crucial for *Bacillus anthracis* Pathogenesis

*Bacillus anthracis*, a gram-positive spore-forming bacterium, is the etiologic agent of anthrax [1,2,3,4]. It is important to note that, outside of the laboratory, the spore is the only infectious form of *B. anthracis* [5,6,7,8]. In most cases of anthrax acquired by inhalation, the spores are thought to be transported from their site of deposition in the lungs to regional lymph nodes where they germinate and outgrow into the vegetative bacilli [9,10,11,12,13,14,15]. The bacilli multiply within the lymph nodes and are then released, rapidly spread systemically, and produce large amounts of the anthrax toxins. Germination near the entry site or in epithelial/nonlymphoidal tissues has also been demonstrated though the significance of these events is uncertain [8,16,17,18,19]. *B. anthracis* spore germination and subsequent disease progression has been reported to be substantially different in cutaneous models of disease [20,21,22]. Nevertheless, evidence exists for toxin production during the earliest stages of spore germination, as will be reviewed.

In a natural setting anthrax is predominantly a disease of herbivores feeding on vegetation from fields contaminated with *B. anthracis* spores. Carnivores can become exposed to/infected by *B. anthracis* when feeding on animals which previously died of anthrax [23,24,25]. In humans, there are three major forms of anthrax as delineated by the route of spore exposure, cutaneous, gastrointestinal and inhalational [26].

The large majority of reported anthrax cases are cutaneous infections. Although cutaneous infections can be fatal, they are more typically self-limited, with mortality rates in untreated cases of about 20% [27,28,29]. A comparatively new form of human disease, injectional anthrax, has been observed among intravenous drug users, *i.e.*, heroin addicts in Western Europe [29,30,31,32,33,34]. This disease can range from cutaneous/intradermal to septicemic anthrax [32]. Gastrointestinal infections in humans have been reported, but are considered rare [35,36,37,38]. The biodefense community is most concerned with the third major form of disease, inhalational anthrax, due to the potential ease of aerosol exposure to lethal doses of spores, non-specific clinical symptoms, and rapid progression; if left untreated it has a mortality rate approaching 100% [39,40]. Thus, early diagnosis of inhalational anthrax can be very challenging and unless there is a high index of suspicion, the disease can rapidly progress to a stage which is no longer treatable with antibiotics. All of these forms of infection are initiated by the introduction of ungerminated spores into the susceptible host. Ultimately, fatal anthrax is the result of acute intoxication and massive bacteremia [39].

The overwhelming effects of anthrax lethal and anthrax edema toxins are well characterized and have been reviewed extensively [13,41,42,43,44,45]. The EF is a calmodulin-dependent adenylate cyclase [46,47] and the LF is a zinc-matalloproteinase known to induce MAPKK cleavage [48,49]. Both the EF and LF require the protective antigen (PA) component, encoded by the *pagA* gene, to facilitate translocation into the host cells cytosol where they can act [50,51,52,53]. Importantly, the PA protein has been shown to elicit a strong and protective immune response and accordingly has served as the primary vaccine antigen in effective human anthrax vaccines [54]. The exact mechanisms and interactions of these toxins and individual toxin components are described in detail in numerous review articles [41,42,43,55,56]. Our focus will be to summarize the interactions of these toxins and toxin components with *B. anthracis* spores. The potential ramifications of these interactions will be described.

## 2. Ungerminated *Bacillus anthracis* Spores Contain Detectable Levels of PA and Are Affected by Anti-PA Antibodies

One of the earliest reports of the phenomena resulting from spore and anti-toxin antibody interactions was published in 1996. Stepanov *et al.* demonstrated that immunoglobulins arising from vaccination with the live attenuated ST-1 vaccine strain had anti-toxin effects as expected, but also had effects on *B. anthracis* spores [57]. These observations suggested that the immune response resulting from such a vaccination could prevent lethal intoxication but also potentially alter the earliest stages of the disease pathogenesis (*i.e*., when the ungerminated spores are first introduced into the host). IgM collected from rabbits vaccinated with ST-1 strain opsonized germinating spores resulting in a significantly increased rate of phagocytosis. Antibodies to the anthrax toxin components also inhibited spore germination, whereas other presumably unrelated antibodies (*i.e*., antibodies induced by *Clostridium botulinum*, *Francisella tularensis*, *Yersinia pestis*, and measles virus) did not inhibit spore germination. They demonstrated that anti-staphylococcal antibodies inhibited germination, but maintained that this finding supported the existence of significant similarities between *B. anthracis* and *Staphylococcus* bacteria. These results clearly set the stage for further characterization of vaccine-induced antibody and spore interactions, in particular the potential ability of anti-toxin antibodies to modify spore germination and subsequent host-interactions. This concept was novel because, based upon previous understanding of the anthrax life cycle, significant amounts of toxin should not be present until vegetative cell replication was well underway, as described in the previous section. It should be noted that since toxin-based vaccines effectively protect animals against infection with *B. anthracis* and not just against intoxication, it follows logically that the toxins (and presumably the immune response to them) would have major roles from the initial stages of infection [58].

Later work clearly demonstrated that ungerminated *B. anthracis* spores contained a detectable level of toxin components (at least PA). This was demonstrated initially by electron microscopy, SDS PAGE gel analyses, and subsequently additional sensitive assays as described below [59,60,61]. Whether this spore-associated PA is an innate product of the spores or merely represents an artifact from sporulation conditions and/or spore purification procedures has not been fully resolved. It was, however, clearly shown that the amount of PA on ungerminated spores was sufficiently adequate to interfere with spore germination and influence opsonization of the spores in *in vitro* macrophage assays performed in the presence of anti-PA antibodies. These antibody interactions were hypothesized to potentially impact the earliest stages of infection soon after the first introduction of spores into the host.

## 3. Anthrax Toxin Components Are Produced by Germinating Spores

In order to initiate disease, ungerminated *B. anthracis* spores that were introduced into the host must germinate and replicate. The transition from ungerminated spore to germinated cell is a complex cascade of events that can occur very rapidly in a suitable environment [62,63,64,65]. An early publication by Guidi-Rontani *et al.*, detailing spore-macrophage interactions early after infection, demonstrated that the LF component of anthrax toxin was produced by germinating spores associated with macrophages within 3 h post-infection [66]. This was the first account demonstrating toxin component production at such an early time point.

Electron microscopy studies have clearly identified an association of PA and germinating spores [61,67]. The morphologically distinct germinated spores appeared to interact less with anti-PA antibodies than ungerminated spores [59]. This observation led to experiments to quantify PA production by germinating spores. Defined minimal germination induction solutions were used in order to induce germination but not allow outgrowth or replication of bacilli. Media used in these studies included AI (*l*-alanine and inosine) and AAC (*l*-alanine, adenosine, and casamino acids) [59]. AI will not permit vegetative growth even after 24 h incubations and AAC medium (more complex/metabolically complete than AI) will not permit replication during short term assay periods [59] but will allow vegetative outgrowth after extended incubations [68]. Spores exposed to AAC medium were lysed and the RNA extracted. The *pagA* transcript was detected by reverse-transcription PCR analysis after only 15 min of exposure to AAC.

Additionally, an immunomagnetic electrochemiluminescense assay (ECL) was employed in these studies to detect PA production using anti-PA antibodies, as described previously [59,69]. ECL utilizes antibodies (in this case a pool of monoclonal anti-PA antibodies) conjugated to microbeads. Detection was then achieved by the addition of ruthenium labeled polyclonal rabbit anti-PA antibodies. It was determined that the limit of detection under these conditions was approximately 100 pg/mL [59]; other similar ECL assays have achieved sensitivities in the femtogram range, demonstrating the very high sensitivity that can be achieved with such detection assays [69]. PA was detected on spores incubated in AAC medium within 60 min. The data acquired using the less nutritive AI medium suggested that PA could be observed on these germinating spores within a 2 h incubation period. The fact that the amount of PA found on the germinating spores appeared to correlate with the complexity of the medium, was further proof that germinating spores can produce detectable levels of toxin components. Supernatants collected from germinating spores were also tested for the presence of PA. Regardless of the medium used, AI or AAC, detectable levels of PA were observed in the supernatants. These findings indicated that spores can potentially synthesize and secrete or release these toxin components during early stages of germination.

## 4. The Interactions among *Bacillus anthracis* Spores, Toxins and Host Macrophages Are Complex

The interactions among spores, toxins and host cells can be complex in nature. The deleterious effects of anthrax toxins on the host immune response have been well documented [55]. In the traditional anthrax paradigm, systemically replicating vegetative bacilli produce dramatically enhanced quantities of anthrax toxins during the later stages of infection. The toxins and/or toxin components interact with anthrax toxin receptors on the cell surface and are subsequently translocated into the cytosol of the cell. To date, there are two known anthrax toxin receptors, TEM8 [70] and CMG2 [71,72]. Other work has documented that the LDL receptor-related protein LRP6 and the β-1 integrin can impact anthrax toxin receptor function and that complexes containing β1-integrin can also act independently as low affinity toxin receptors [73,74]. Anthrax toxins have been shown to negatively impact macrophages [75,76,77,78,79,80,81,82,83,84], neutrophils [85,86,87], dendritic cells [20,88,89,90], and other host cell types [91,92,93]. In 2005, Banks *et al.* presented a model which suggests that after phagocytosis of spores by host cells (*i.e*., macrophages), the spore can germinate and begin producing toxins within the phagolysosome [94]. Anthrax toxin receptors can be located on the inside of the phagolysosomes [94,95], and thus are available to interact with the toxins secreted by the newly germinated spores. The toxin components LF and EF are then translocated into the cell cytosol, resulting in intoxication by the germinating spores from within the macrophage and the subsequent downstream events described above. Thus, this model highlighted alternate interactions that could potentially take place among spores, toxins and host cells.

It was previously demonstrated that macrophages were important in limiting the ensuing anthrax infection in a mouse model of anthrax. In these studies mice that were chemically depleted of macrophage populations were significantly more susceptible to anthrax initiated by the intraperitoneal injection or inhalation of spores [67,96]. Conversely, mice that received supplementary treatments with tissue culture macrophage-like cells (RAW264.7 cells) or mice that received starch as an eliciting agent for native macrophage recruitment were significantly more likely to survive challenge with lethal doses of spores [96]. Furthermore, it was demonstrated that treatment with RAW264.7 cells harboring an inactivated anthrax toxin receptor, protected the mice from *B. anthracis* infection at rates even greater than did treatment with wild-type RAW264.7 cells [97], further documenting the interplay between spore infections and intoxications. Specifically these results appear to demonstrate both the susceptibility of macrophages to the lethal effects of toxin produced by the germinating organisms and paradoxically the ability of macrophages to protect the host against *B. anthracis* infection.

Hanna *et al.* demonstrated that macrophage depletion results in mice becoming more resistant to intoxication. In these studies mice were challenged with anthrax lethal toxin alone and did not initiate an infection [98]. This report suggested a role for macrophages in facilitating infection or intoxication (*i.e*., the Trojan horse concept) [14,15,98]. Collectively these data as well as more recent studies may contribute towards explaining the basis of the long-observed inverse correlation between sensitivity to infection and to toxin challenge in some animals [99,100]. The presence of increased numbers of macrophages might protect better against spore infection but increase sensitivity to toxin. The potential role(s) of other host cells types and other bacterial factors in anthrax mortality remains to be completely elucidated and is beyond the scope of this review.

Thus, taken together, these data demonstrate several scenarios that might be observed during the pathogenesis of anthrax. It is important to note that these situations are rarely synchronous and their potential outcomes can vary depending on many factors including strain of mouse, strain of *B. anthracis*, challenge route/site, or cell type(s) studied. Additionally, even in the same model system (*i.e*., *in vitro* macrophage assay) there can be variables which can alter the outcome. Such variables include the number of spores phagocytosed by the cell, and the proportions of spores which germinate after phagocytosis and which survive the anti-bacterial environment of the phagolysosome [7,97]. For example, in a single macrophage with a relatively heavy spore burden, some spores may remain ungerminated, others may germinate and succumb to killing prior to outgrowth, and another subpopulation may germinate (potentially causing intoxication of the macrophage), outgrow, and escape from the cell.

Antibodies directed against spore-specific antigens have been shown to be opsonic [101,102,103,104]. Similar effects of anti-PA antibodies on the spore macrophage interactions have also been documented. In tissue culture assays, anti-PA antibodies are opsonic and significantly augment the rate of spore phagocytosis by macrophages as well as enhance the sporicidal activity of the macrophages. These studies were performed using ungerminated spores as well as germinated spores. Interestingly, the opsonic and augmented killing effects were generally lost approximately 24 h after spore germination, further supporting the concept that spores rapidly release PA upon germination [60,61]. An additional and separate spore effect attributed to anti-PA antibodies has been the demonstration that anti-PA antibodies inhibit germination of *B. anthracis* spores *in vitro*. This was demonstrated by using a semi-automated fluorescence-based germination assay, by showing antibody-mediated inhibition of spore stainability in the presence of germinant, and by examining spore refractility using phase contrast microscopy [59,105]. The significance of these *in vitro* and *in situ* antibody-mediated effects in the immune responses of the host animal to spore challenge remains to be determined.

Our goal in this brief review has been to highlight the complexity as well as the importance of spore, toxin, and host interactions. Anti-toxin antibodies undoubtedly play an essential role in protecting vaccinated individuals from a lethal anthrax infection. However, the data reviewed herein also help to reinforce the concept that these anti-PA antibodies (and conceivably anti-LF or anti-EF antibodies) likely play a role in infection control very soon after the introduction of spores into the host. Because of emerging and engineered threats it is crucial to continue investigating novel vaccine strategies to protect the warfighter and potentially civilian populations. A better understanding of these early interactions may hold the key to such strategies.
